# Supplementary material for: Adverse Effects of Aβ1-42 Oligomers: Impaired Contextual Memory and Altered Intrinsic Properties of CA1 Pyramidal Neurons
Source: Biomolecules. 2024 Nov 8;14(11):1425. doi: 10.3390/biom14111425 (PMC11591707; doi:10.3390/biom14111425)
Supplement: Supplementary file 1 [file biomolecules-14-01425-s001.zip › biomolecules-3253629-Supplementary information Min dm6.pdf]

Supplementary Information

Title: Adverse effects of $A\beta_{1-42}$ oligomers: impaired contextual memory and altered intrinsic properties of CA1 pyramidal neurons

Authors: Min-Kaung-Wint-Mon[1], Kida H[1], Kanehisa I[1], Kurose M[1], Paw-Min-Thein-Oo[1], Ishikawa J[1], Sakimoto Y[1], Kimura R[2], and Mitsushima D[1,3,*].

Affiliations: [1]Department of Physiology, Yamaguchi University Graduate School of Medicine, Yamaguchi, 755-8505, Japan.
[2]Center for Liberal Arts and Sciences, Sanyo-Onoda City University, Sanyo-Onoda, Yamaguchi, 756-0884, Japan.
[3]The Research Institute for Time Studies, Yamaguchi University, Yamaguchi, 753-8511, Japan.

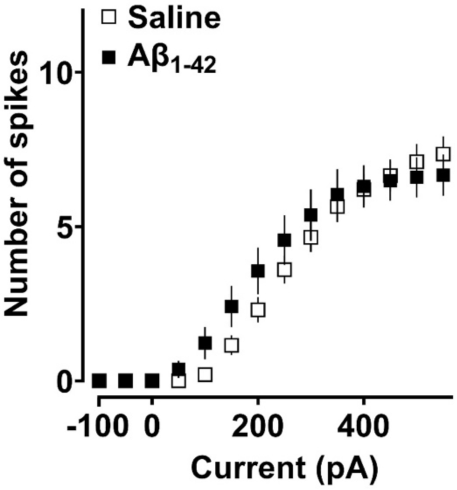

Figure S1. Input-output relationship in saline and $A\beta_{1-42}$ oligomer-injected rats without IA task. Two-way repeated measures ANOVA showed no main effects of oligomers ($F_{1,630} = 2.578$, $p = 0.109$) or interaction with current ($F_{13,630} = 0.640$, $p = 0.821$), but only the main effect of current was observed ($F_{13,630} = 53.732$, $p < 0.0001$). Saline; n = 20 neurons, $A\beta_{1-42}$; n = 27 neurons. Data are expressed as mean ± SEM.

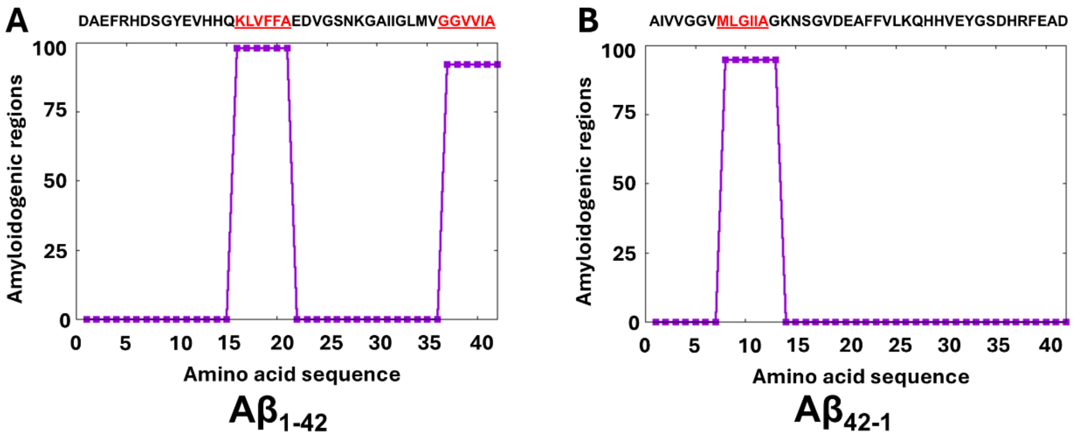

Figure S2. The amyloidogenicity of the amino acid sequence of the $A\beta_{1\text{-}42}$ peptide and its reverse peptide, $A\beta_{42\text{-}1}$. Plots were generated using WALTZ (Waltz (switchlab.org)), where the x-axis represents the amino acid sequence and the y-axis represents the amyloidogenic regions. The WALTZ algorithm identifies two peaks indicating two amyloidogenic regions (residues 16–21 and residues 37–42) in the $A\beta_{1\text{-}42}$ peptide (A), whereas only one region of the $A\beta_{42\text{-}1}$ peptide between residues 8 and 13 has amyloidogenic potential (B).

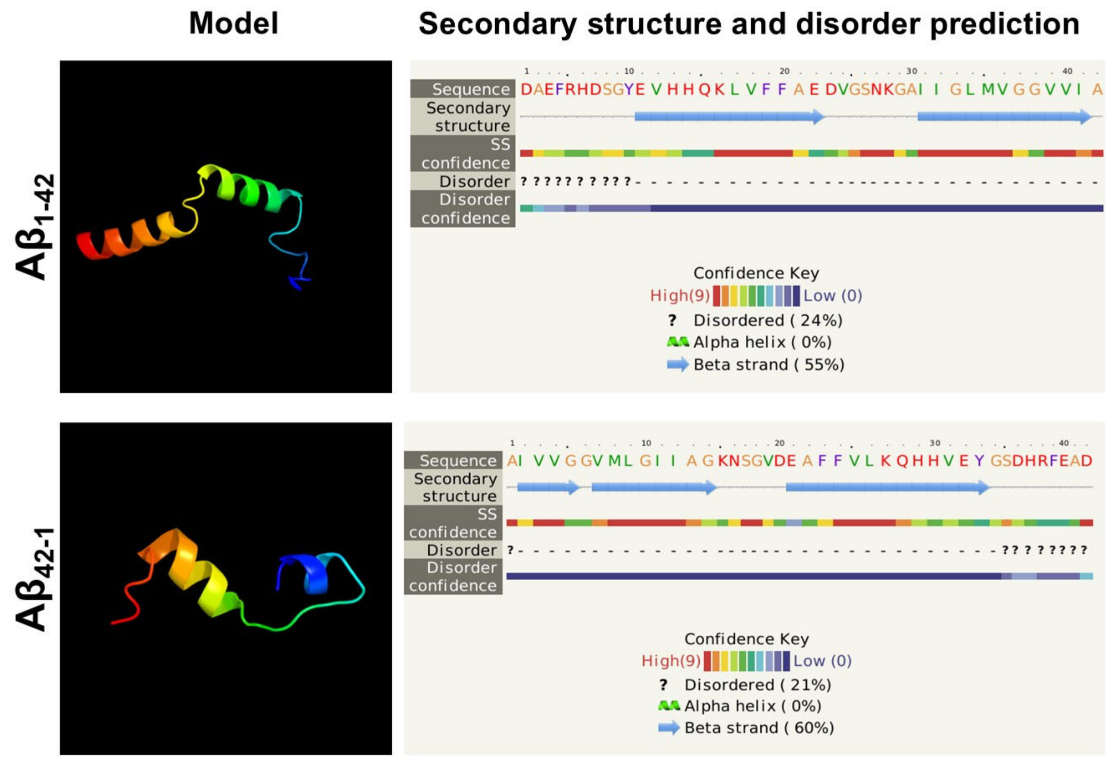

Figure S3. The predicted structure view and amino acid sequence analysis of the Aβ$_{1-42}$ peptide and its reverse peptide, Aβ$_{42-1}$. The predicted secondary structure and sequence profile graph were obtained using the Phyre2 web portal based on the previous protocol [79: http://www.sbg.bio.ic.ac.uk/phyre2]. The images were rainbow colored from N-terminus to C-terminus (left panels) and the model dimensions (Å) were X: 31.568, Y: 27.304, Z: 42.065 for the Aβ$_{1-42}$ peptide and X: 21.848, Y: 18.205, Z: 33.122 for the Aβ$_{42-1}$ peptide. In addition, 42 residues (100% of the Aβ$_{1-42}$ sequence) were modeled with 99.9% confidence, but only 28 residues (67% of the Aβ$_{42-1}$ sequence) were modeled with 5.2% confidence. The secondary structure and disorder prediction graphs (right panels) show that more β-strands were predicted to form in Aβ$_{42-1}$ with comparable disordered sequences.

Table S1. Intrinsic properties of CA1 pyramidal neurons in saline and Aβ1-42 oligomer-injected rats without IA task.

Membrane properties	Saline	Aβ1-42	*p* value
Cm (pF)	184.60 ± 18.32 (22)	206.8 ± 15.5 (31)	0.4957
Rm (MΩ)	88.79 ± 3.96 (22)	134.92 ± 9.97 (31)	0.0111*
Tau (ms)	2.31 ± 0.27 (22)	1.95 ± 0.18 (31)	0.1910
RMP (mV)	-62.53 ± 0.65 (22)	-64.42 ± 0.47 (31)	0.0593
Threshold potential (mV)	-34.21 ± 0.91 (22)	-35.18 ± 0.76 (31)	0.5314
Rheobase (pA)	188.64 ± 12.28 (22)	164.52 ± 8.35 (31)	0.1790

Data are expressed as mean ± SEM. The number of cells is indicated in parentheses. Statistical analysis was performed by unpaired *t*-test.

Table S2. Exploration time of novel-to-be and familiar objects in the sampling phase of an object recognition task.

Group	Novel-to-be (s)	Familiar (s)	*p* value
Saline	21.75 ± 3.10 (8)	22.88 ± 2.55 (8)	0.7200
Aβ1-42	17.23 ± 2.53 (13)	18.38 ± 2.70 (13)	0.8175
Aβ42-1	17.09 ± 2.69 (11)	20.36 ± 3.30 (11)	0.3255

Data are expressed as mean ± SEM. The number of rats is indicated in parentheses. Statistical analysis was performed by paired *t*-test.

Table S3. Total exploration time of familiar and novel objects in the test phase of the object recognition task.

Parameter	Saline	Aβ1-42	Aβ42-1
Total time (s)	33.88 ± 6.50 (8)	31.15 ± 4.09 (13)	26.00 ± 3.94 (11)

Data are expressed as mean ± SEM. The number of rats is indicated in parentheses. No significant changes were found by one-way ANOVA.

Table S4. Total exploration time of familiar and novel social targets in the test phase of the social recognition task.

Parameter	Saline	$A\beta_{1-42}$	$A\beta_{42-1}$
Total time (s)	73.13 ± 8.10 (8)	79.92 ± 11.79 (13)	95.55 ± 8.14 (11)

Data are expressed as mean ± SEM. The number of rats is indicated in parentheses. No significant changes were found by one-way ANOVA.

References

79. Kelley, L. A., Mezulis, S., Yates, C. M., Wass, M. N., & Sternberg, M. J. E. (2015). The Phyre2 web portal for protein modeling, prediction and analysis. *Nature Protocols*, *10*(6), 845–858. https://doi.org/10.1038/nprot.2015.053.
